# Lea’s Series of Pocket Textbooks

**Published:** 1907-03

**Authors:** 


**Lea's Series of Pocket Textbooks.** Diseases of Children. By **George M. Tuttle,** M.D., Attending Physician to St. Luke's Hospital, St. Louis, Mo. 2d edition, revised. 12mo, 392 pages, with 5 plates. Edited by **Bern. B. Gallaudet,** M.D. Lea Brothers & Co, Philadelphia and New York, 1907. (Cloth, $1.50; flexible leather, $2.00; net prices).

This is one of the most important of the Lea Pocket Textbook Series and the superior excellence of Tuttle's presentation of the subject makes it one of the more valuable of the smaller work on pediatrics. Only those diseases and phases of physical disturbance which are met with in the period from birth to adolescence are considered, and the author has presented in a very compact and understandable volume all the essentials of an important branch of medicine.

